# A distal intergenic region controls pancreatic endocrine differentiation by acting as a transcriptional enhancer and as a polycomb response element

**DOI:** 10.1371/journal.pone.0171508

**Published:** 2017-02-22

**Authors:** Joris van Arensbergen, Sebastien Dussaud, Corinne Pardanaud-Glavieux, Javier García-Hurtado, Claire Sauty, Aline Guerci, Jorge Ferrer, Philippe Ravassard

**Affiliations:** 1 Genomic Programming of Beta-Cells Laboratory, IDIBAPS, Barcelona, Spain; 2 CIBER de Diabetes y Enfermedades Metabólicas, Barcelona, Spain; 3 Sorbonne Universités, UPMC Univ Paris 06, Inserm, CNRS, Institut du cerveau et de la moelle (ICM)–Hôpital Pitié-Salpêtrière, Boulevard de l’Hôpital, Paris, France; 4 Department of Medicine, Imperial Centre for Translational and Experimental Medicine, Imperial College, London, United Kingdom; University of Bremen, GERMANY

## Abstract

Lineage-selective expression of developmental genes is dependent on the interplay between activating and repressive mechanisms. Gene activation is dependent on cell-specific transcription factors that recognize transcriptional enhancer sequences. Gene repression often depends on the recruitment of Polycomb group (PcG) proteins, although the sequences that underlie the recruitment of PcG proteins, also known as Polycomb response elements (PREs), remain poorly understood in vertebrates. While distal PREs have been identified in mammals, a role for positive-acting enhancers in PcG-mediated repression has not been described. Here we have used a highly efficient procedure based on lentiviral-mediated transgenesis to carry out in vivo fine-mapping of, cis-regulatory sequences that control lineage-specific activation of *Neurog3*, a master regulator of pancreatic endocrine differentiation. Our findings reveal an enhancer region that is sufficient to drive correct spacio-temporal expression of *Neurog3* and demonstrate that this same region serves as a PRE in alternative lineages where *Neurog3* is inactive.

## Introduction

Embryonic development involves the establishment of progressively divergent transcriptional programs. This process relies on the dynamic activation of developmental regulatory genes, which is largely determined through the interaction of lineage-specific transcriptional activators with cis-acting sequences located in enhancers [1,2].

Strict spatiotemporal regulation is further determined by mechanisms that repress lineage-specific regulatory genes. Polycomb group (PcG) proteins are thought to play a major role in the repression of such genes [3–5]. PcG-dependent repression is mediated by two major complexes known as PRC1 and PRC2 that act in an interdependent manner. PRC2 contains Ezh2 and Ezh1 in its non-canonical form, both of which catalyzes the trimethylation of Lysine 27 on histone 3 (H3K27me3). This histone mark contributes to the recruitment of the PRC1 complex, which promotes H2A ubiquitylation, interference with transcriptional machinery, and chromatin compaction [4–8].

In contrast to the extensive knowledge of how DNA binding factors are recruited to specific genomic regions to promote lineage-specific transcription, the recruitment of PcG complexes remains poorly understood. In *Drosophila*, Polycomb response elements (PREs) are enriched in specific DNA binding factor motifs, and can therefore often be predicted [9]. To demonstrate an element functions as a PRE, it must be shown that it can autonomously recruit polycomb proteins outside of its normal genomic environment, which has been done for several Drosophila PREs [9,10]. In vertebrates, our understanding of PREs is more limited, and so far no combination of DNA-binding motifs has been able to predict their existence. Recent studies, however, have shown that PcG proteins primarily target GC-rich promoter regions, and suggest that a high density of unmethylated CpGs may be sufficient to promote PcG recruitment and repression in the absence of activating signals [11–14]. Other work however suggests that CpG islands are not required for the recruitment of PcG- proteins in the HoxD gene cluster *in vivo* [15]. In addition to promoter associated PREs, distal PREs have been identified in the mammalian genome, adding to the challenge of understanding what characterizes a PRE and how they contribute to gene regulation [13,15–17]. Thus, current evidence implicates both CpG-rich promoter-proximal regions and distal regions in the recruitment of PcG repressive complexes. However, enhancer regions exerting a dual regulatory function of promoting transcription in one lineage and ensuring PcG-mediated repression in another lineage, have not been shown.

In the current study we have examined the cis-regulatory mechanisms of *Neurog3*, which encodes a transcription factor that is essential for the formation of all pancreatic endocrine lineages, including insulin-producing β-cells [18–20]. *Neurog3* is transiently activated in a subpopulation of cells of the ductal epithelium of the embryonic pancreas, and orchestrates a lineage-committed progenitor program that leads to the differentiation of hormone-producing cells [19–22]. Understanding the mechanisms that control *Neurog3* activation is therefore critical for efforts to artificially program functional β-cells.

Previous work demonstrated that the 5.7 kb upstream sequence of human *NEUROG3* can drive a correct spatio-temporal expression pattern in transgenic mice [23], yet critical cis-elements within this sequence remain to be established. Several studies have revealed transcription factors that interact with specific sequences in the *Neurog3* 5’ region [23–26], although the *in vivo* function of such regulatory elements has not been examined.

So far, studies of the transcriptional mechanisms that underlie pancreas development have largely focused on activating transcription factors and cis-regulatory elements [23–29]. Pancreatic cell differentiation, however, is also linked to dynamic changes in PcG-repressed chromatin [30–32], although nothing is known about the sequence elements that direct the repressive programs that are relevant for pancreatic endocrine differentiation.

Here we used an efficient lentiviral transgenesis strategy to dissect the function of *Neurog3* cis-regulatory elements *in vivo*. Using this approach, we identify an enhancer region that is sufficient to direct correct expression of the *Neurog3* gene in embryonic pancreatic progenitors, and in this enhancers we map discrete cis-elements that are recuired for its function. In addition, our study shows that the same enhancer region acts as a PRE in alternate cellular lineages, thereby illustrating how one genomic region can exert a dual function as an enhancer and as a PRE.

## Results

### An enhancer region that activates *Neurog3* in endocrine progenitors

**Fig 1:**
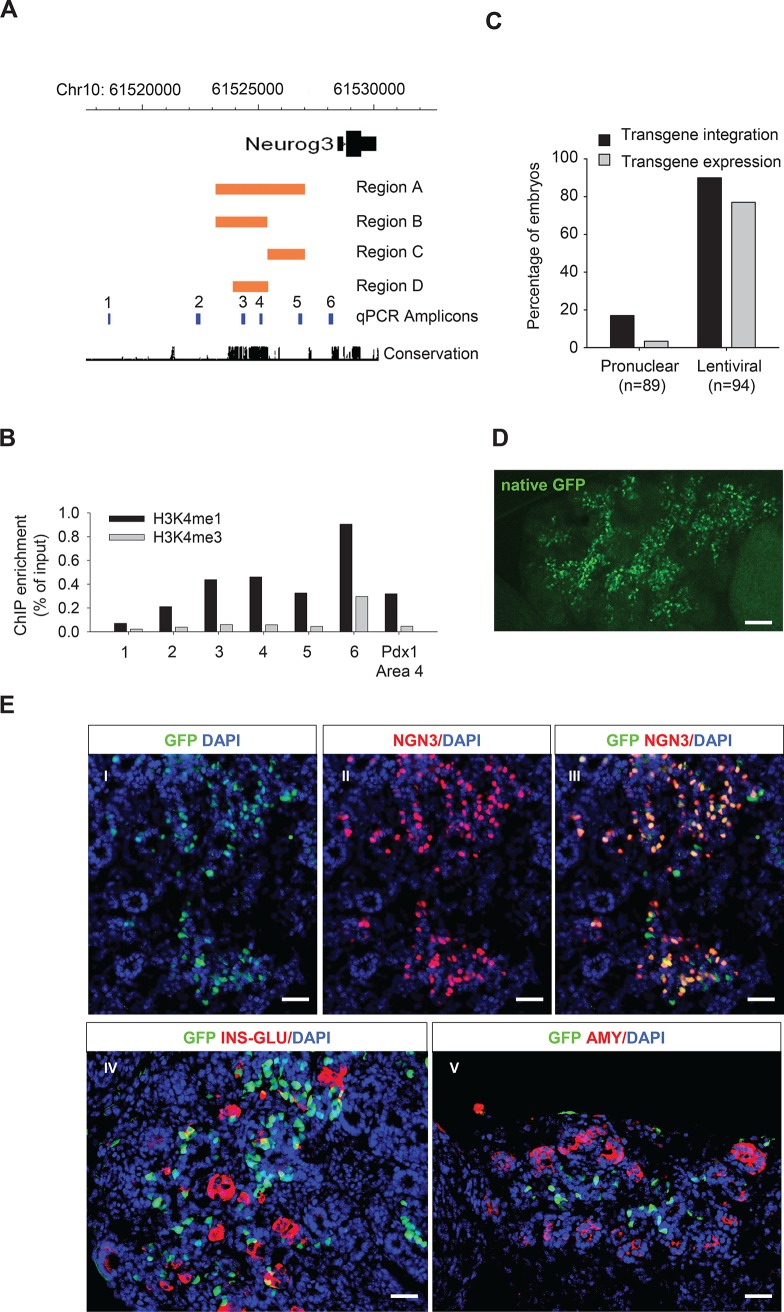
Mapping a *Neurog3* enhancer region through highly efficient lentiviral transgenesis. **A.** Schematic representation of the screened *Neurog3* locus. Orange bars depict regions A-D that were selected for characterization through standard transgenesis. PCR amplicons that were used for ChIP analysis (in **B**) are shown in blue. The conservation track represents conservation over 17 vertebrate species. **B**. ChIP revealed H3K4me1 without H3K4me3 enrichment in E13.5 pancreatic buds in putative *Neurog3* enhancer regions (n = 1; note that the H3K4me3 signal results from Neurog3+ cells which represent <5% of the pancreatic bud). Pdx1 Area 4 is shown as a positive control as it represents a known enhancer. **C**. Lentiviral integration increases the efficiency of transgenesis and transgene expression compared with standard pronuclear injections. **D, E.** Expression of GFP under the control of the *Neurog3* enhancer region B in lentiviral transgenics. Native GFP in a whole mount follows the trunk of E14.5 pancreas (**D**) and coincides with endogenous Neurog3 in (**E**, panel I-III), whereas no overlap is seen with Amylase (red; panel V) and only rare overlap is seen with Insulin and Glucagon (panel IV). Occasional GFP-positive Neurog3-negative cells were found at E14.5, most of which belonged to the endocrine lineage (panel III, IV). Scale bars are 75 μm (**D**) and 50μm (**E**).

Sequence conservation and monomethylated Histone 3 Lysine 4 (H3K4me1) in the absence of trimethylated H3K4 (H3K4me3) are known hallmarks of transcriptional enhancers [33]. To identify sequences that regulate the expression of *Neurog3* during development, we screened the *Neurog3* 5’ region for evolutionary sequence conservation and H3K4me1-enrichment in mouse pancreatic bud from embryonic day 13.5, when *Neurog3* expression peaks. Conservation was particularly high in the region stretching from approximately -5kb to -3kb relative to the transcription start site (TSS) (Fig 1A). This same region also showed H3K4me1 enrichment in the absence of H3K4me3, to a similar extent as a well-known pancreatic enhancer of the *Pdx1* gene (Fig 1B) [34].

Based on these findings we first tested the ability of a fragment stretching from approximately -5.5 kb to -1.5 kb of the *Neurog3* gene to drive expression of a reporter gene selectively in Neurog3+ cells using standard transgenesis (S1 Fig). This 3.9kb element (construct A; Fig 1A) was able to reproduce selective expression of the LacZ reporter gene in all Neurog3+ cells, namely the ventral hypothalamus, the ventral spinal cord, the duodenum and the pancreas, with only minimal ectopic expression (S1A Fig). To assess if a similar result could be obtained by a smaller fragment, we generated construct B, C and D (Fig 1A) and demonstrated that only construct B (representing the 5 prime 2.2kb of construct A) consistently reproduced the expression pattern of *Neurog3* with the exception of expression in the ventral spinal cord (S1B Fig). These results demonstrate that enhancer region B, hereafter referred to as the *Neurog3* enhancer region, contains all the regulatory sequences to drive correct spatio-temporal expression of *Neurog3* in the pancreas.

### Recapitulation of *Neurog3* enhancer activity using highly efficient lentiviral-mediated transgenesis

Next we aimed to further dissect the *Neurog3* enhancer region by generating deletions of putative regulatory elements. However, the efficiency of standard transgenesis is prohibitively low to allow for a comprehensive screen of this nature. For example, in the abovementioned analysis, 263 embryos were collected, of which only 38 (14.5%) showed transgene integration and only 3 of them expressed LacZ in the pancreas at embryonic day 14.5. We thus tested lentiviral transduction in fertilized eggs followed by embryo implantation in pseudopregnant mice as an alternative strategy to produce transgenic animals [35]. After initial transduction with a lentiviral vector expressing GFP under the control of the *Neurog3* enhancer region, we confirmed transgene integration in 85 out of 94 (90.4%) embryos and transgene expression in 72 (76.6%) embryos. In comparison, after pronuclear injection of the same *Neurog3* enhancer region, only 3 out of 89 (3.4%) collected embryos expressed the transgene (Fig 1C). Taken together, the data indicate that in our hands lentiviral transgenesis is at least 20-fold more efficient than standard transgenesis (Fig 1C). In lentiviral transgenic embryos, selectivity of GFP expression in Neurog3+ cells was similar to the results obtained through standard transgenesis (Fig 1D and 1E, S1 and S2 Figs). Having thus validated lentiviral transgenesis for the assessment of enhancer function, we set out to identify critical activating elements within the *Neurog3* enhancer region.

### A 50 bp cis-element is essential for Neurog3 enhancer activation in pancreatic progenitors

**Fig 2:**
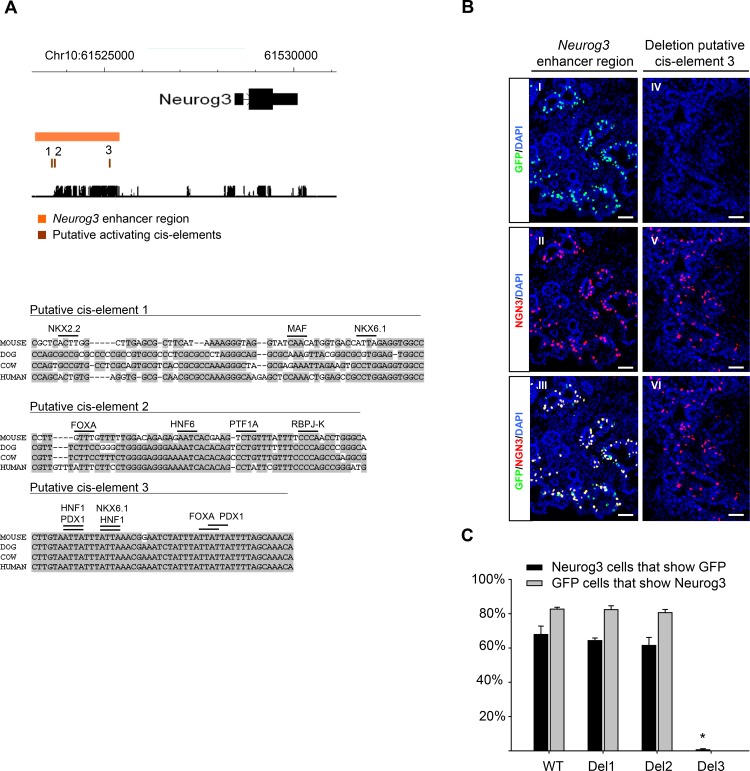
An essential activating cis-element within the *Neurog3* enhancer region. **A.** Three elements in the *Neurog3* enhancer displayed clustered binding motifs for known pancreatic transcription factors. Black lines above sequence indicate 4 bp core of transcription factor motif. **B.** Deletion of cis-element 3 (Del3; but not 1 or 2) from the *Neurog3* enhancer region leads to a near complete loss of expression. The panels show GFP (green) and Neurog3 (red) in an E14.5 transgenic for the wild-type *Neurog3* enhancer region (panel I-III) and a transgenic for the *Neurog3* enhancer region with activating cis-element 3 deleted (panel IV-VI). **C.** Quantification of percentage of Neurog3 cells that are GFP positive (black bars) and the percentage of GFP cells that are Neurog3 positive (grey bars; not present for Del3 because in 7 out of 8 embryo’s no GFP cells were detected. Quantification was done on 8 embryos for each genotype in which slides amounting to at least 1000 Neurog3 cells were counted ([*] *P* = 5.3 x 10^−9^, Student’s t-test with Bonferroni correction). Scale bars: I-VI = 50 μm.

As a first step to identify critical activating sequence elements within the *Neurog3* enhancer region, we searched for ~50 bp elements enriched in binding site sequence motifs for pancreatic transcription factors. We identified 3 motif clusters of 50–65 bp length located ~ -4.9 to -3.3kb upstream of the *Neurog3* TSS, which we refer to as candidate cis-elements 1, 2 and 3 (Fig 2A). We assessed if these elements were essential for *Neurog3* expression by generating lentiviral-mediated transgenic animals with the *Neurog3* enhancer region carrying deletions for each of these 3 cis-elements. While loss of element 1 or 2 did not change the expression pattern in any of the transgenic embryos (16 and 17 transgenics studied, respectively), loss of element 3 led to a full abrogation of expression in 28 out of 30 transgenic embryos (Fig 2B and 2C). A critical role for this same element (previously described as ‘Cluster 1’ in human [23]) had been suggested previously based on interactions with key pancreatic transcription factors, although its *in vivo* function was not tested [23,26]. Taken together, these results uncover a distal 50 bp cis-element that is essential for activation of the *Neurog3* enhancer in embryonic pancreatic endocrine progenitors.

### Pdx1, Foxa2 and Hnf1b act on cis-element 3 to activate *Neurog3*

**Fig 3:**
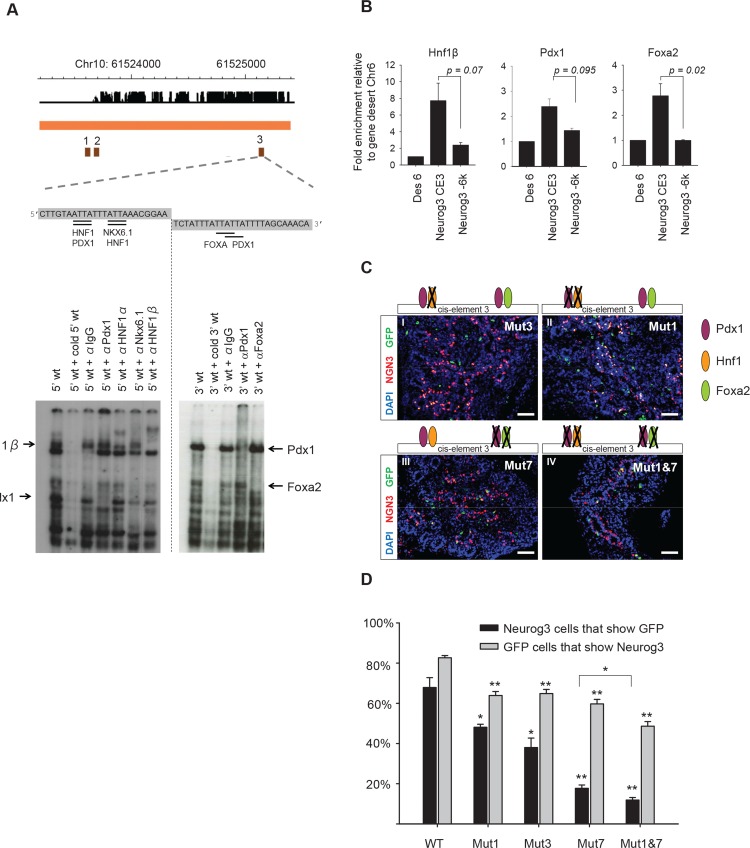
Identification of cis-regulatory mutations that disrupt activation of *Neurog3* in pancreatic progenitors. **A.** Binding of Hnf1b, Pdx1 and Foxa2 to cis-element 3 of the Neurog3 enhancer using EMSAs with nuclear extracts from E13.5 pancreatic buds. The 5’ part of the cis-element 3 sequence binds Hnf1b and Pdx1 while the 3’ part binds Pdx1 and Foxa2. **B.** ChIP for Hnf1b (n = 3), Pdx1 (n = 2) and Foxa2 (n = 3) in E13.5 pancreatic buds. Binding to the cis-element 3 (*Neurog3 CE3*) was compared to control regions *Des6* (locus in gene-desert) and *Neurog3 -6K* (*Neurog3 5’* upstream region). Indicated *P*-values were calculated with Students t-test. **C.** Lentiviral transgenesis using enhancers with indicated mutations, all of which disrupt specific transcription factor binding sites (**S**3 **Fig**). Note how the strongest reduction of expression is found upon disruption of all identified binding sites within cis-element 3. **D.** Quantification of percentage of Neurog3 cells that are GFP positive (black bars) and the percentage of GFP cells that are Neurog3 positive (grey bars). Quantification was done on 8 embryos for each genotype in which at least 1000 Neurog3 cells were counted ([**] *P*<1.0 x 10^−6^, [*] *P*<0.02, Student’s t-test with Bonferroni correction). Scale bars: C I-VI = 50 μm.

The analysis of transcription factor binding motifs together with previous work suggested that the *Neurog3* enhancer region exerts its activating effect through the binding of Pdx1, Foxa2 and Hnf1b to cis-element 3 [23,26] (Fig 2A). To address this hypothesis we first tested binding of these factors to cis-element 3 using EMSA. Using nuclear extracts from pancreatic buds isolated at E13.5 and specific antibodies we confirmed binding of Pdx1, Foxa2, and Hnf1b (Fig 3A). Next, we performed ChIP on E13.5 pancreatic bud chromatin. This experiment, while technically challenging as <5% of the cells in pancreatic buds express *Neurog3*, suggested that these three factors also bind to the cis-element 3 of the endogenous enhancer region *in vivo* (Fig 3B).

To further address the activating role of this regulatory element we designed mutations that selectively disrupt binding of Hnf1 (Mut3), or both Pdx1 and Hnf1 (Mut1) to the 5’ side of cis-element 3, or else binding of both Pdx1 and Foxa2 to the 3’ side of cis-element 3 (Mut7) (S3 Fig, S1 Table and S2 Table). In lentiviral-mediated transgenic animals, all three mutations, and most clearly Mut7, caused a reduction in the expression penetrance (number of Neurog3 cells that show GFP) and cell-type specificity of expression (number of GFP cells that express Neurog3, Fig 3C and 3D). Next, we combined Mut1 and Mut7 and found a further 33% reduction in the combined mutant as compared to Mut7 alone for the number of Neurog3 cells that express GFP (Fig 3C and 3D). These results suggest that Pdx1, Foxa2 and Hnf1b activate *Neurog3* through cis-element 3 in a partially redundant manner. Importantly, the data identifies discrete nucleotides that are essential to for the ability of the Neurog3 enhancer to drive expression in the embryonic pancreas *in vivo*.

### The *Neurog3* enhancer represses transcription in alternate lineages

**Fig 4:**
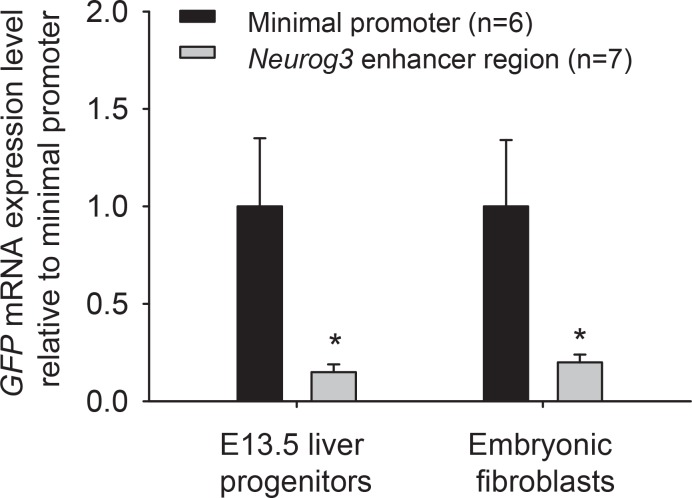
The *Neurog3* enhancer region contributes to transcriptional repression in alternative lineages. qRT-PCR analysis showed that in E13.5 liver progenitors and embryonic fibroblasts from transgenics carrying the Neurog3 enhancer, GFP mRNA was significantly reduced. GFP transcript levels were normalized for *Tbp* and compared to the construct that lacked the Neurog3 enhancer region ([*] (*P*<0.05, Student’s t-test).

**Fig 5:**
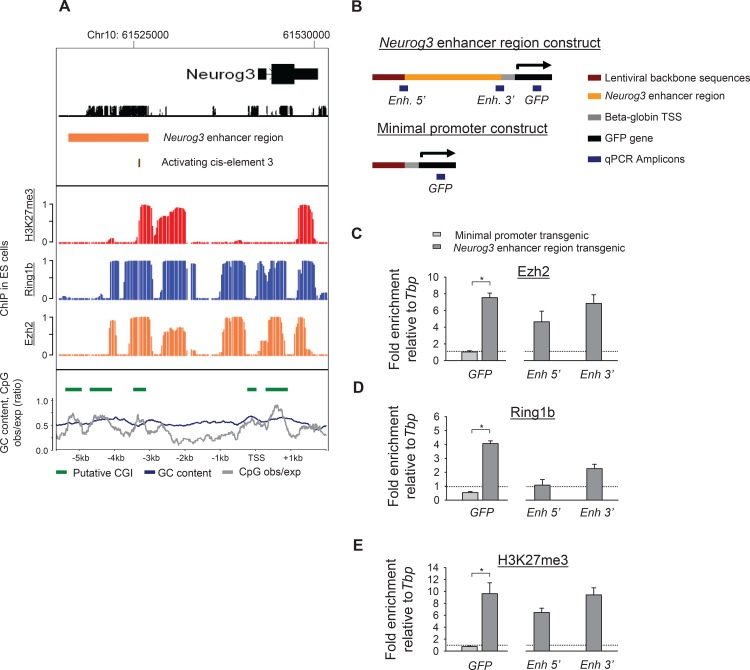
The minimal *Neurog3* enhancer functions as a PRE *in vivo*. **A.** ChIP-oligonucleotide array analysis of the endogenous mouse *Neurog3* locus in ES cells. The graph shows significance of enrichments for H3K27me3 (red), Ring1b (blue), and Ezh2 (orange) in posterior probability ranging from 0 to 1. The bottom panel shows a CpG content analysis for the same genomic coordinates. The average GC content in 400 bp sliding windows is shown in blue and the CpG fold over expected is shown in grey. Green bars depict putative CpG islands. **B.** Schematic representation of amplicons used in ChIP-qPCR to selectively study the integrated exogenous *Neurog3* enhancer region or the control minimal promoter (note that only GFP can be used on both). **C-E.** ChIP-qPCR analysis for Ezh2, Ring1b and H3K27me3 on the exogenous *Neurog3* enhancer region in transgenic E13.5 liver (n = 2) ([*] *P*<0.01, Student’s t-test).

Next, we assessed whether the *Neurog3* enhancer region, in addition to its ability to selectively drive expression in endocrine progenitors, could also contribute to transcriptional repression in non-expressing lineages. We therefore isolated transgenic mouse embryonic liver progenitors from E13.5 transgenic embryos that carried GFP under the control of the minimal promoter with or without the *Neurog3* enhancer region and studied GFP expression. As expected, GFP was not detectable by fluorescence microscopy in both groups. However, low levels of transcript were detectable by qPCR, and they were 5-fold lower in transgenics that carried the *Neurog3* enhancer region than in trangenics that integrated the backbone vector containing only the minimal promoter and GFP (Fig 4, see Fig 5B for a schematic representation of the two constructs). Similar results were obtained in transgenic mouse embryonic fibroblasts in the presence of the *Neurog3* enhancer region (Fig 4). This effect did not result from differences in the number of integration events of the two constructs (S4 Fig). These results fit the notion that in addition to its ability to drive expression in endocrine progenitors, the Neurog3 enhancer region can exert transcriptional repression in alternative developmental lineages.

### The *Neurog3* enhancer region functions as a PRE *in vivo*

Next, we investigated whether the *Neurog3* enhancer region induces transcriptional repression in alternative cellular lineages by functioning as a PRE. To address this, we first analyzed H3K27me3 enrichment in cell types that do not express *Neurog3* including Pdx1 positive purified E10.5 pancreatic progenitor and adult islets that represent developmental stages that precede and follow Neurog3 expression, respectively. We observed H3K27me3 within and around the endogenous enhancer region in virtually all cell types analyzed [30] (S5 Fig). Moreover, we performed ChIP for the PcG subunits Ezh2 and Ring1b in ES cells and found these to be enriched in and around the *Neurog3* enhancer region (Fig 5A). It is known that PcG-mediated repression preferentially occurs among CpG-island containing genes, and recently it was proposed that CpG islands themselves play a role in recruiting PcG proteins [12,13]. Consistent with this notion, the *Neurog3* enhancer region contains several putative CpG-rich areas (Fig 5A). These findings suggested that this enhancer region might act as a PRE, although the same results could arise if other sequences that are adjacent to the *Neurog3* enhancer region are responsible for recruiting PcG subunits to the enhancer region. We thus tested if transgenes that carried the *Neurog3* enhancer region are capable of recruiting PcG subunits Ezh2 and Ring1b and can establish H3K27me3-enriched chromatin in liver from E13.5 embryos. We first examined embryonic liver cells from lentiviral transgenics that lacked the enhancer region, and found that they did not exhibit H3K27me3 enrichment and were not bound by PcG proteins at the transgenic locus (Fig 5C–5E). By contrast, in transgenic animals that integrated the *Neurog3* enhancer region, the exogenous transgene locus was bound by Ezh2 and Ring1b and displayed H3K27me3 enrichment that stretched into the GFP coding region (Fig 5C–5E). To assess the role of cis-element 3 in the establishment of PcG-mediated repression we performed ChIP for H3K27me3 in animals that were transgenic for the *Neurog3* enhancer region with a deletion of cis-element 3. The results demonstrated that the 50 bp cis-element was dispensable for the establishment of H3K27me3 enrichment (S6A Fig). Finally, we tested whether the *Neurog3* enhancer promotes PcG-mediated repression that extends into adulthood, and found that indeed H3K27me3 enrichment was present in pancreatic exocrine tissue and skeletal muscle from adult transgenic mice (S6B and S6C Fig). Taken together, these data demonstrate that the minimal *Neurog3* enhancer region functions as a PRE *in vivo*.

## Discussion

The exact manner in which discrete genomic sequences provide either activating or repressive instructions to ensure lineage-specific gene transcription is still incompletely understood. Here we applied an efficient lentiviral transgenic strategy to understand the cis-regulatory sequences that underlie the initiation of the pancreatic endocrine differentiation program. Our data revealed a distal *Neurog3* enhancer region that serves as a platform through which activating transcription factors direct *Neurog3* expression in the pancreatic epithelium. This same enhancer region simultaneously acts as a PRE in alternate developmental lineages. Thus, we have identified a genomic region that functions as a lineage-specific enhancer and as a PRE.

### A highly efficient transgenic approach to dissect cis-regulatory function

Understanding the cis-regulatory mechanisms that underlie organogenesis and differentiation is a fundamental challenge for biomedical research. Current epigenomic assays theoretically allow for high-throughput prediction of genomic regulatory elements. For example, active enhancers can be predicted by the combinatorial presence of specific histone modifications, nucleosome depletion, or transcription factor occupancy patterns [36–38]. Understanding the function of such predicted elements requires experimental analysis in meaningful developmental contexts. Existing technologies for this purpose, however, remain inefficient. Zebrafish provide an efficient model system to test putative regulatory elements, but it remains unproven that most mammalian cis-regulatory elements are appropriately recognized in this species. A significant example of a divergent regulatory mechanism in zebrafish is that although they develop an endocrine pancreas, the putative orthologue of *Neurog3* is not expressed in endocrine progenitors [39]. Given their evolutionary proximity and extensive validation, mouse transgenics remain a desirable model system to study mammalian cis-regulatory mechanisms [37].

We have here used lentiviral mediated transgenesis to increase the throughput of mouse transgenesis for the discovery and fine mapping of *Neurog3* regulatory sequences. This approach enhanced the efficiency of transgene expression by approximately 20-fold relative to conventional transgenesis, without compromising the specificity of expression. The results suggest that mouse lentiviral transgenesis is a robust tool for discovery, fine-mapping, and characterization of mammalian regulatory sequences, and can thus prove useful to elucidate the role of cis-regulatory elements in development and disease.

### Dissection of an enhancer that recapitulates pancreatic *Neurog3* expression

We have identified a Neurog3 enhancer region that is sufficient to selectively drive expression in Neurog3+ multipotent ductal epithelial cells, and have fine-mapped a critical cis-element in this enhancer. Although our studies are the first to define this enhancer region and demonstrate the critical role of a ~50bp cis-element *in vivo*, a critical role for this cis-element had been proposed previously based on the fact that it was bound by key pancreatic regulators [23,24,26]. Within this activating sequence we performed a mutation screen to further finemap the critical bases. Notably, two of the mutations that inhibit *Neurog3* activation were high-affinity binding sites for Pdx1, a transcription factor previously shown to be a critical regulator of *Neurog3* activation and of the specification of pancreatic endocrine progenitors [26]. We also identified a functionally important binding site for Hnf1b, which plausibly mediates the role of this transcription factor in pancreatic endocrine specification [40]. Other cis-elements and transcription factor binding sites were found to be functionally redundant, underscoring the importance of functional assessment of such regulatory elements *in vivo*. These findings can be exploited to address how *Neurog3* becomes activated in the multipotent pancreatic epithelium, and to efficiently target prospective pancreatic endocrine progenitors.

### The *Neurog3* enhancer region functions as a PRE *in vivo*

The demarcation of sharp lineage boundaries of gene expression is directed by cell specific DNA-binding activators as well as by repressive mechanisms. Distal regulatory regions play a pivotal role by providing binding sites for cell-specific transcriptional activators, whereas the recruitment of repressive complexes to specific genomic sites is less understood.

Recently it was proposed that in mammals, unlike current knowledge based on studies in *Drosophila*, the main factor determining the recruitment of PcG complexes is the presence of non-methylated GC-rich elements [12,13]. This has supported a model in which GC-rich promoter regions are repressed by default and become activated upon clearing of PcG repression through local or distal binding of activating transcription factors [12,13,41,42]. Our experiments have now revealed an enhancer region that not only activates its target gene in a lineage-selective manner, but also recruits PcG-mediated repression in non-expressing developmental lineages. While previous studies in mammals had shown PREs that do not overlap promoters [13,15,17] and even presence of H3K27me3 at poised enhancers [43,44], the findings described here are of interest in that they reveal that an enhancer can direct both lineage-specific transcriptional activation and PcG-mediated repression. Deletion experiments, nevertheless, demonstrated that the sequences required for activation were distinct from the sequences required for PcG-mediated repression. Further studies should explore the prevalence of this developmental cis-regulatory mechanism in metazoan genomes.

## Methods

### Mouse models

All experiments were approved by the Institutional Animal Care Committee of the University of Barcelona or by the Direction Départementale de Protection des Populations (DDPP) de Paris, under accreditation number A75-13-19.

Animals were sacrificed by cervical dislocation according to the recommended practices of the French legislation.

### DNA constructs for conventional transgenesis

A HindIII–HindIII fragment of the 5’flanking region of Neurog3 located between nucleotide -5284 and -1428 relative to transcription start site was amplified by PCR using Phusion DNA polymerase (Finnzyme) a high fidelity thermostable DNA polymerase and the following primers: -5284 sense AAGCTTTGTGTGGAAGGA and -1428 antisense AAGCTTCTAGTACGTTCTAACT. The amplification was performed on a lambda clone containing a 20kb fragment of the Neurog3 mouse locus. The resulting PCR product was digested by HindIII and cloned into the HindIII site of the pBGZ40 reporter plasmid upstream a human beta globin minimal promoter linked to *lacZ and a SV40 poly adenylation signal* [45]. The resulting construct-A was entirely sequenced to rule out undesired mutations and further digested by XhoI and XbaI prior to pronuclear injection. A similar approach was used to generate constructs-B, -C and–D using the following primers. Construct-B: -5284 sense and -3061 antisense AAGCTTGCTAGCATTGCCTGGGGG. Construct-C: -3057 sense TTTACCCCCTCCCAACAG and -1428 antisense. Construct-D -4916 AAGCTTCCAGCCATAAGGTTTATT and -3061 antisense.

### DNA constructs for lentiviral transgenesis

The construct-B PCR fragment was cloned into the pENTR/D-TOPO vector (Invitrogen) according to the manufacturer’s instructions to generate the 2.2 kb wild type enhancer entry clone. A destination vector containing a Gateway recombination cassette rfa followed by the pBGZ40 beta globin minimal promoter linked to eGFP was constructed in the pTRIP lentiviral backbone using a similar strategy as described [46]. The 2.2 kb enhancer was inserted into the lentiviral destination vector by in vitro gateway recombination using LR clonase II following manufacturer’s instructions. Note that all lentiviral vectors containing cis element deletions or specific point mutations were all cloned into the lentiviral destination vector using the same gateway recombination procedure.

### Cis-element deletions and site directed mutagenesis

Deletions and point mutations were all performed on the pENTR 2.2 kb enhancer plasmid using a PCR based mutagenesis system. Briefly, sense and antisense complementary primers overlapping either deletion area or containing the point mutations were designed. Such mutation containing primers were used for a first round of PCR with two external 5’ and 3’ primers to generate two PCR products. The left product was obtained using the mutated primer antisense along with the 5’ external primer and the right PCR product with the sense mutated primer and the 3’ external primer. Both PCR products were generated using Phusion DNA polymerase (Finnzyme) and no more than 25 PCR cycles. Both DNA fragments were purified on Wyzard PCR cleanup columns (Promega) and 1ng of each resulting purification were used as matrix for a second round of PCR using the 2 external primers. The resulting PCR products contained the desired deletions or point mutations and were further digested with BstEII and PstI for deletions of cis-element 1 and 2 or by SpeI and NcoI for all other deletions or point mutations. The digested fragments were cloned into the pENTR 2.2 kb enhancer plasmid digested with BstEII and PstI or SpeI and NcoI to replace a wild type cassette by a mutation containing one. Note that the double mutation 1–7 was generated using mutation 1 as original matrix with the PCR primers of mutation 7. The complete list of mutation primers and external primer is presented in **S2 Table**. For constructs with deletions of cis-element 1, 2 or 3 the deleted fragments are located from nucleotide -4877 to -4812 (relative to transcription start site), -4877 to -4812pb and -3336 to– 3287 respectively.

### Conventional transgenesis

Transgenic embryos were generated by pronuclear injection into fertilized eggs from FVB/NRj mouse strain (Elevage Janvier). The injected eggs were next re-implanted into F_1_ hybrids (DBA/2 × C57Bl6) pseudo pregnant females. Most constructs were assayed in founder (F_0_) transgenic embryos with the exception of construct-A for which 2 different mouse transgenic lines have being generated.

### Production of lentiviral vectors

Lentiviral vector stocks were produced as previously described [47]. The amount of p24 capsid protein was quantified by the HIV-1 p24 ELISA antigen assay (Helvetica Health Care). All transductions of fertilized eggs were performed using normalized titers relative to p24 capsid protein quantification ranging between 50 to 60 ng of p24 per microliter of viral stock.

### Lentiviral transgenesis

Transgenic embryos were generated by injection of lentiviral vectors into the perivitelline space of fertilized eggs from RjOrl:SWISS mouse strain as described previously [48]. The injected eggs were next re-implanted into F_1_ hybrids (DBA/2 × C57Bl6) pseudo pregnant females. All constructs were assayed in founder (F_0_) transgenic embryos.

### Genotyping of transgenic animals

Genomic DNA was extracted from either tail of adult mice or yolk sac of embryos using proteinase K digestion followed by PCR amplification to detect LacZ or GFP transgenes with the following primers. LacZ1: 5’ ACCCTGGCGTTACCCAACTTAATCG 3’; LacZ2: 5’ ACAAACGGCGGATTGACCGTAATGG 3’; GFP sense: 5’ GACCACATGAAGCAGCACGACTTCT 3’; GFP antisense: 5’ TTCTGCTGGTAGTGGTCGGCGAGCT 3’.

### Derivation of embryonic fibroblast culture from transgenic embryos

E14.5 transgenic embryos containing either the Neurog3 enhancer region or the minimal promoter GFP construct were collected. For each collected transgenic embryo a separate fibroblast culture was derived. Briefly, a small part of the skin of the embryo was collected under sterile condition and placed in a 24 well culture plate under cover glass and culture at 37°C under 5% CO2 for 2 days in culture medium containing 10% FCS 1% essential amino acids (Invitrogen), 1% Penicillin and Streptomycin (Invitrogen) in DMEM 4.5 g/l D-glucose (Invotrogen). After 2 days, the skin explant was removed and the fibroblasts were passaged by Trypsin / EDTA treatment in a 6 well plate. When confluence was observed cells were passaged into a 6 cm diameter plate and next into a 10 cm diameter plate. Cells were amplified subsequently by passages at confluence with a 1 to 5 dilution until frozen or used.

### RNA extraction

Total RNA was extracted using RNeasy micro kit (Qiagen) followed by DNAase I treatment. RNA integrity was verified with a 2100 Bioanalyzer (Agilent) and reverse transcribed using Superscript 2 (invitrogen).

### Quantitative PCR analysis

Quantitative PCR of reverse-transcribed RNA, ChIP or gDNA samples was performed on a 7300 Realtime PCR System (Applied Biosystems) using the Power SYBR Green reagent (Applied Biosystems). Quantities were determined using 2^(-Delta Delta C(T))^. A full list of primers used is provided as **S3 Table**.

### Tissue preparation for histological analysis

Transgenic embryos were collected at different stages. The date of reimplantation of the one cell stage embryos in pseudo-pregnant females was considered as embryonic day 0.5 (E0.5). Embryos were dissected and a tissue block containing the stomach; pancreas and duodenum was removed and fixed by immersion in 4% PFA for 24 h. Tissues were cryoprotected by incubation for 48 h in 15% sucrose prepared in PBS and next embedded in 15% sucrose, 7% gelatin prepared in PBS then frozen in isopentane at -50°C. 10 μm sections were performed using a cryostat (Leica). Whole embryos were fixed in 4% PFA for 20 min and stained for *lacZ* reporter activity as described and embedded in BSA-gelatin [49]. 300-μm sections were cut with a Vibratome (Leica VT1000S) as described [50].

### Immunostaining

10μm cryosections were used for immunofluorescence staining as described previously [51]. The following primary antibodies were used: rabbit anti-insulin (1/1500 Euromedex Ref. 20056), rabbit anti-glucagon (1/1500 Euromedex Ref. 20076), rabbit anti-amylase (1/350 Sigma Ref. A8273) mouse anti-Neurog3 (1/1000 Beta Cell Biology Consortium Ref. AB2013) and chicken anti-GFP (1/1000 Antibodies-Online GMBH Germany, Ref. ABIN 147441). The corresponding secondary antibodies were used: Alexa 488 Goat anti Chicken (1/2000 Life Technologies Ref. A11039) and Alexa 555 Goat anti Rabbit (1/1000 Life Technologies Ref. A21428). Neurog3 detection was amplified as described by Zahn et al. 2004 [52]. TSA amplification (Perkin Elmer TSA-Direct NEL702) was preformed prior to GFP staining. Beta-galactosidase was detected by immuno-staining using rabbit anti-βGal (1/1000 Cappel Inc.) and stained with the Vectastain DAB kit (Vector Inc.) as described [53].

### ISH

Riboprobes were synthesized from various cDNAs inserted into pGEM-T Easy vector (Promega). After linearization of the plasmids, non-radioactively labeled riboprobes were synthesized in the presence of 3.5 nmol of either digoxigenin (Dig)-11-UTP or fluorescein (Fluo)-12-UTP (Roche) by using the riboprobe combination system Sp6/T7 (Promega). The LacZ riboprobe spans the whole coding sequence. The Neurog3 and Insulin riboprobes were described previously [50,54]. Hybridization experiments were performed essentially as described previously on 10μm cryosections [55]. For double in situ staining probes were detected with alkaline phosphatase-conjugated anti-Dig and anti-Fluo antibodies (Roche). Colorimetric detection was achieved with 5-bromo-4-chloro-3-indolylphosphate (BCIP) and either nitroblue tetrazolium (NBT; Promega) or 2-(4-iodophenyl)-3-(4-nitrophenyl)-5-phenyltetrazolium chloride (INT; Roche). Dig- and Fluo-labeled riboprobes were applied together to the slides. After detection of the Dig-labeled probe with NBT-BCIP, the anti-Dig antibody was removed by washes with 0.1 M glycine (pH 2.2)-0.1% Tween before incubation with the anti-Fluo antibody. No background signal was observed on slides hybridized with the sense control probes and incubated with NBT-BCIP for at least as long as the corresponding antisense probes.

### Quantification of Neurog3 and GFP expression in transgenic embryos

Transgenic embryos at E14.5 generated with all lentiviral constructs (wild type enhancer, deletions of cis-element 1, 2 and 3 and point mutations 1, 3, 7 and 1–7) were analyzed. GFP + cells, Neurog3 + cells and GFP/Neurog3 double + cells were counted on successive 10μm sections located every 50 μm. A total number of at least 1000 Neurog3 positive cells were counted per embryo. 8 transgenic embryos were thus analyzed for each transgenic construct.

### Chromatin Immunoprecipitation (ChIP)

ChIPs were performed as described [30]. In short, mouse tissues or mouse ES cells (CGR8; [56]) were fixed in 1% formaldehyde for 10 min after which nuclei were purified and sonicated using Bioruptor (Diagenode) to a length of 200 to 1000 bp. Samples were precleared with protein A+G-Sepharose (1:1) and immunoprecipitated with rabbit anti-Ring1b [57], rabbit anti-H3K27me3 (Upstate, 07–449), mouse anti-Ezh2 [58] overnight at 4°C. Immune complexes were collected by adsorption to protein A+G-Sepharose for 2 hr at 4°C. Beads were washed and immunocomplexes eluted prior to DNA purification with Qiaquick columns (Qiagen). Precipitations on pancreatic buds were performed with at least 20 pancreatic buds per IP and in the presence of 2.5 mg/mL BSA and 25 mg/mL tRNA. For tiling array experiments, ChIP and input DNA were amplified as described previously using the Sigma GenomePlex WGA2 kit while adding dUTPs to a final concentration of 0.4 mM during the amplification reaction to enable subsequent fragmentation [30]. We fragmented 6–7.5 μg DNA, labeled it using the Affymetrix GeneChip WT Double-Stranded DNA terminal Labeling Kit, and hybridized to GeneChip® Mouse Promoter 1.0R Arrays.

### EMSA

Single-stranded oligonucleotides (**S1 Table**) were annealed and labeled with [γ-32P]ATP (GE healthcare Rediprime labeling kit). The labeled oligonucleotides were column-purified (GE microspin G-50 columns). 32P-labeled oligonucleotides were incubated for 20 min at room temperature with nuclear extracts in 20mM Hepes (pH 7.9), 90 mM KCl, 5 mM MgCl2, and 0.05% Nonidet P-40, 1mg/ml BSA, 5% Glycerol, 1mM DTT, 1mg/ml poly(dI-dC).poly(dI-dC) and 0.1mM ZnCl. For supershifts, antibodies or preimmune serum was added to the reaction and incubated for 20 min on ice before incubation with the labeled probe. Samples were electrophoresed on a 5% acrylamide gel in 0.5 X TBE buffer and autoradiographed. For competition assays, the ratio of labelled probe to cold competitor was 1:100. The antibodies were Rabbit anti-mouse IgG (Abcam; ab6709), goat anti-Pdx1 (abcam; ab47383), goat anti-Foxa2 (santa-cruz; SC-6554), goat anti-Hnf1b (santa-cruz; SC-7411) and rabbit anti-Hnf1a [59].

### Statistical and integrated data analysis

For GeneChip ChIP experiments enrichment relative to input DNA was determined using Cisgenome [60]. We applied a Hidden Markov Model as described [61].

CpG island analysis was done using CpGPlot in EMBOSS [62]. We scanned the *Neurog3* region with a 400 bp sliding window in steps of one base to calculate GC content and the ratio of observed CpGs and expected CpGs (CpG (obs/exp)) for each centred position. The cut-off for CpG (obs/exp) and GC content was set to an average of 0.6 and 0.5 respectively in a set of 10 windows. Furthermore we required an island to be at least 100 bp in length.

Motif search was performed on the complete Neurog3 enhancer region using free access version of Genomatix Matinspector with the default settings [63]. The obtained output was manually searched for known pancreatic factors. For the selected sites the lowest Core similarity was 0.767 and the lowest matrix similarity was 0.744. These results were manually curated based on more recent ChIP-seq based motifs where available.

All barplots are shown as means with error bars representing the SEM. Comparisons were done as indicated with Student’s two-sample, two-sided t-test and Bonferroni corrections were done for the number of comparisons.

### Data access

Microarray data for ChIP experiments of Ezh2 in ES cells will be made publicly available through ArrayExpress under accession number E-MTAB-1460 (reviewer password: nxgEEa62). Other data sets have been described before [30,31].

## Supporting information

S1 Fig **Regions A and B recapitulate the *Neurog3* expression pattern in conventional trangenics. A.** LacZ expression under the control of enhancer region A (see **Fig 1** for representation of enhancer segments). In E11.5 embryos (panel I-IV), region A recapitulated the previously reported pattern of expression of *Neurog3* in the spinal cord (SC) that displays 2 stripes, the medial stripe (MS) and the ventral stripe (VS) (panel I), the ventral hypothalamus (vHyp; panels II, III) and the pancreatic bud (PB; panel IV) with ectopic expression only in the cephalic mesenchyme (CM; panel III) and the notochord (NC; panel III). In panel IV the stomach (St) and the liver (L) are indicated. At E14.5 LacZ was observed in the pancreas (P; panel V) and the duodenum (D; panel VI) **B.** LacZ expression in the E15.5 pancreas from transgenics carrying enhancer region B. LacZ immunostaining show that LacZ (panel II) follows the expected distribution detected by double in situ hybridization of Neurog3 in the pancreatic epithelial tree (blue; panel I, adjacent section to II), whereas insulin (brown; panels I and III) shows the expected distribution in more peripheral regions. Panel III shows and enlargement of the inset in panel I. Panel IV shows an in situ hybridization co-staining for LacZ (brown) and Neurog3 (blue) with an enlargement of the inset shown as panel V. Scale bars: **A** I, II = 200μm, III = 400μm, IV-VI = 200μm, **B** I, II = 100μm, III- V = 25 μm
(TIF)

S2 Fig E18.5 expression of GFP under the control of the *Neurog3* enhancer region in lentiviral transgenics. Expression of GFP was analyzed in the pancreases of E18.5 transgenic embryos. GFP expression (green) was compared to Neurog3 expression (red upper panel) and also compared to Insulin (red left lower panel) and Glucagon (red right lower panel). GFP-positive Neurog3-negative cells were in the endocrine lineage. This became more prevalent at E18.5 compared to E14.5, presumably because of the different stability of GFP and Neurog3. Scale bars: 50 μm.
(TIF)

S3 Fig Disruption of TF binding sites by mutations in cis-element 3. The 5’ part of cis-element 3 (5’WT) was screened with overlapping substitutions of 3 wild-type bases by G nucleotides. The effect of these mutations on DNA binding affinities was assessed by incubating the unlabeled mutant probes at 100-fold excess relative to the wild-type labeled sequence in Min6 nuclear extracts. Note that mutation 1 was found to disrupt both Pdx1 and Hnf1b binding, while Mutation 3 selectively disrupted Hnf1b binding. For the 3’ part of cis-element 3 (3’WT) two mutations were designed and tested in E13.5 pancreatic bud extracts. Both mutations (mut7, mut8) were found to disrupt Pdx1 binding, and mut7 moderately affected Foxa2 binding.
(TIF)

S4 Fig qPCR analysis of transgenic copy numbers for the minimal promoter and the *Neurog3* enhancer region constructs. qPCR was performed on genomic DNA isolated from embryonic tails using the GFP 3’UTR primer pair, and *Cdx2* was used to normalize the data. Each cross represents an animal. The horizontal lines represent the means. N.S., not significant, Student’s t-test.
(TIF)

S5 Fig The *Neurog3* enhancer region shows H3K27me3 enrichment in all non-expressing tissues analyzed. The graph shows the results of an oligonucleotide tiling array analysis of ChIPs for H3K4me3 and H3K27me3 as reported [30]. Enrichment values for H3K4me3 (green) and H3K27me3 (red) are expressed as posterior probability values ranging from 0 to 1.
(TIF)

S6 Fig PcG-mediated repression by the Neurog3 enhancer region is independent of cis-element 3 and extends into adulthood. ChIP analysis of H3K27me3 in the exogenous *Neurog3* enhancer region with or without cis-element 3 in E13.5 liver (**A**), adult acinar tissue (**B**), or adult skeletal muscle (**C**).
(TIF)

S1 Table EMSA double stranded oligonucleotide probe sequences. (DOCX)

S2 Table PCR oligonucleotide primer sequences used for cis-element deletions. (DOC)

S3 Table qPCR oligonucleotide primer sequences for reverse transcription and ChIP experiments. (DOCX)
