# Supplementary figures and images for: A distal intergenic region controls pancreatic endocrine differentiation by acting as a transcriptional enhancer and as a polycomb response element
Source: PLoS One. 2017 Feb 22;12(2):e0171508. doi: 10.1371/journal.pone.0171508 (PMC5321433; doi:10.1371/journal.pone.0171508)

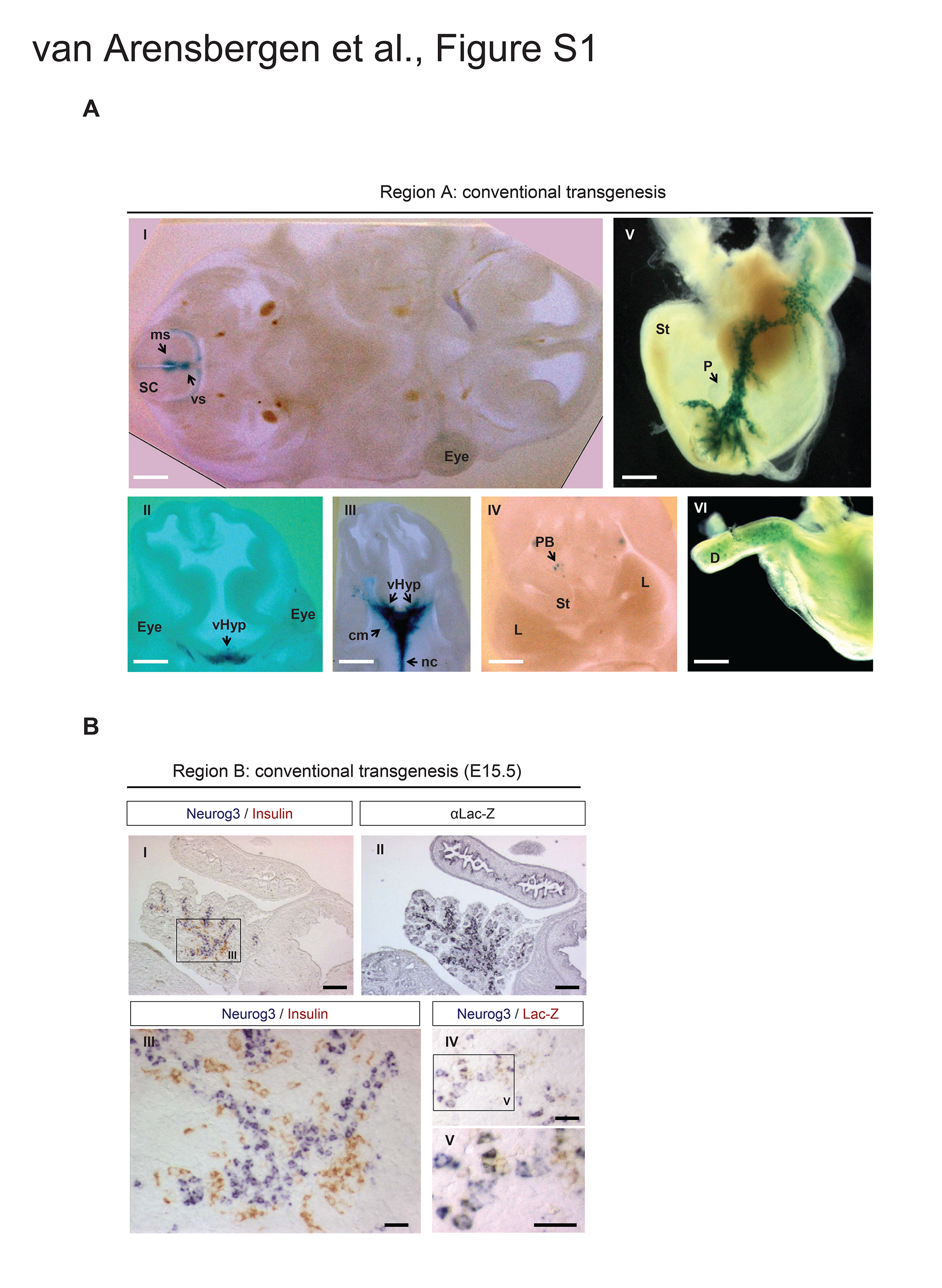

Supplement: S1 Fig — Regions A and B recapitulate the Neurog3 expression pattern in conventional trangenics. A. LacZ expression under the control of enhancer region A (see Fig 1 for representation of enhancer segments). In E11.5 embryos (panel I-IV), region A recapitulated the previously reported pattern of expression of Neurog3 in the spinal cord (SC) that displays 2 stripes, the medial stripe (MS) and the ventral stripe (VS) (panel I), the ventral hypothalamus (vHyp; panels II, III) and the pancreatic bud (PB; panel IV) with ectopic expression only in the cephalic mesenchyme (CM; panel III) and the notochord (NC; panel III). In panel IV the stomach (St) and the liver (L) are indicated. At E14.5 LacZ was observed in the pancreas (P; panel V) and the duodenum (D; panel VI) B. LacZ expression in the E15.5 pancreas from transgenics carrying enhancer region B. LacZ immunostaining show that LacZ (panel II) follows the expected distribution detected by double in situ hybridization of Neurog3 in the pancreatic epithelial tree (blue; panel I, adjacent section to II), whereas insulin (brown; panels I and III) shows the expected distribution in more peripheral regions. Panel III shows and enlargement of the inset in panel I. Panel IV shows an in situ hybridization co-staining for LacZ (brown) and Neurog3 (blue) with an enlargement of the inset shown as panel V. Scale bars: A I, II = 200μm, III = 400μm, IV-VI = 200μm, B I, II = 100μm, III- V = 25 μm (TIF) [file pone.0171508.s001.tif]

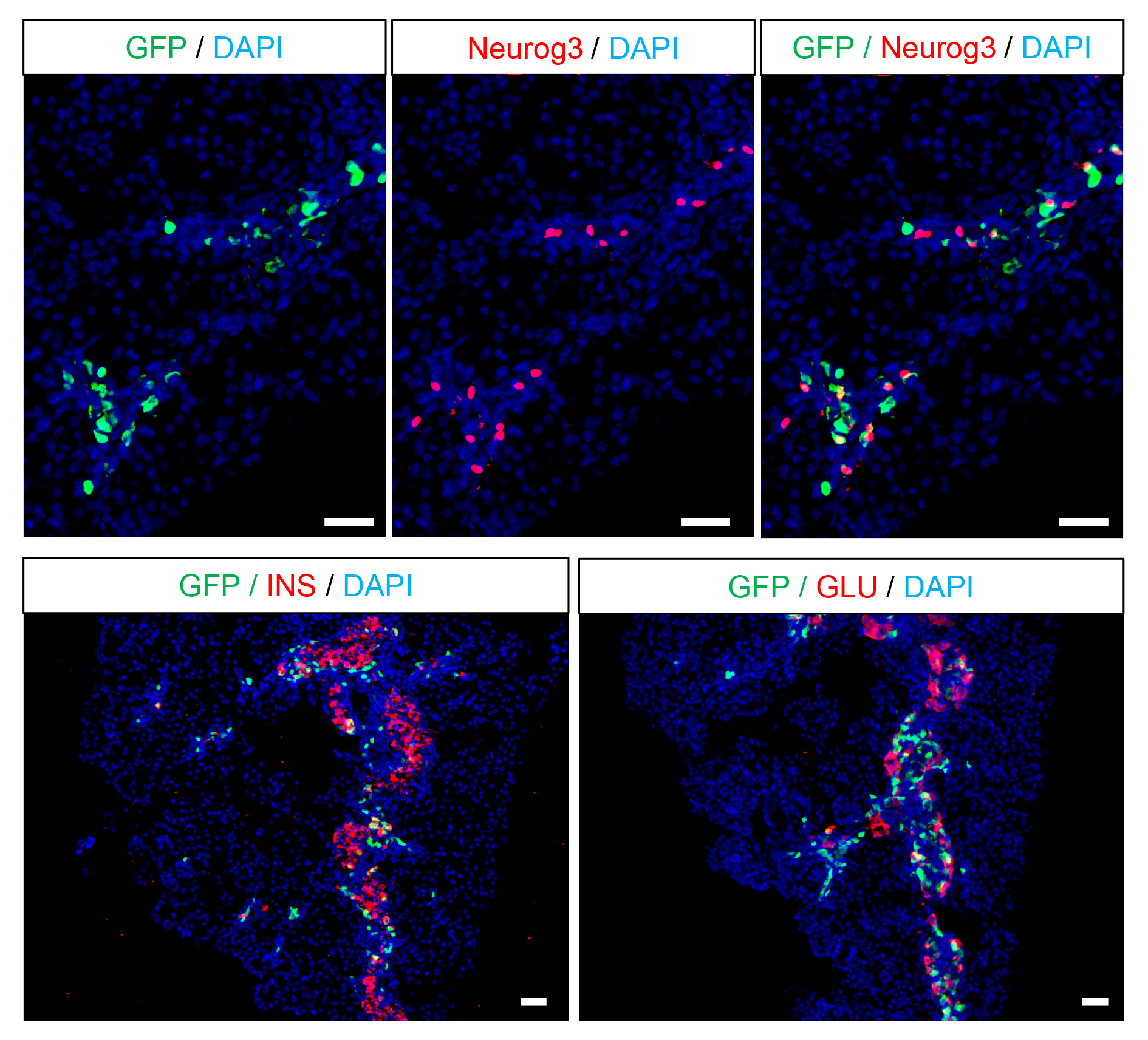

Supplement: S2 Fig — Expression of GFP was analyzed in the pancreases of E18.5 transgenic embryos. GFP expression (green) was compared to Neurog3 expression (red upper panel) and also compared to Insulin (red left lower panel) and Glucagon (red right lower panel). GFP-positive Neurog3-negative cells were in the endocrine lineage. This became more prevalent at E18.5 compared to E14.5, presumably because of the different stability of GFP and Neurog3. Scale bars: 50 μm. (TIF) [file pone.0171508.s002.tif]

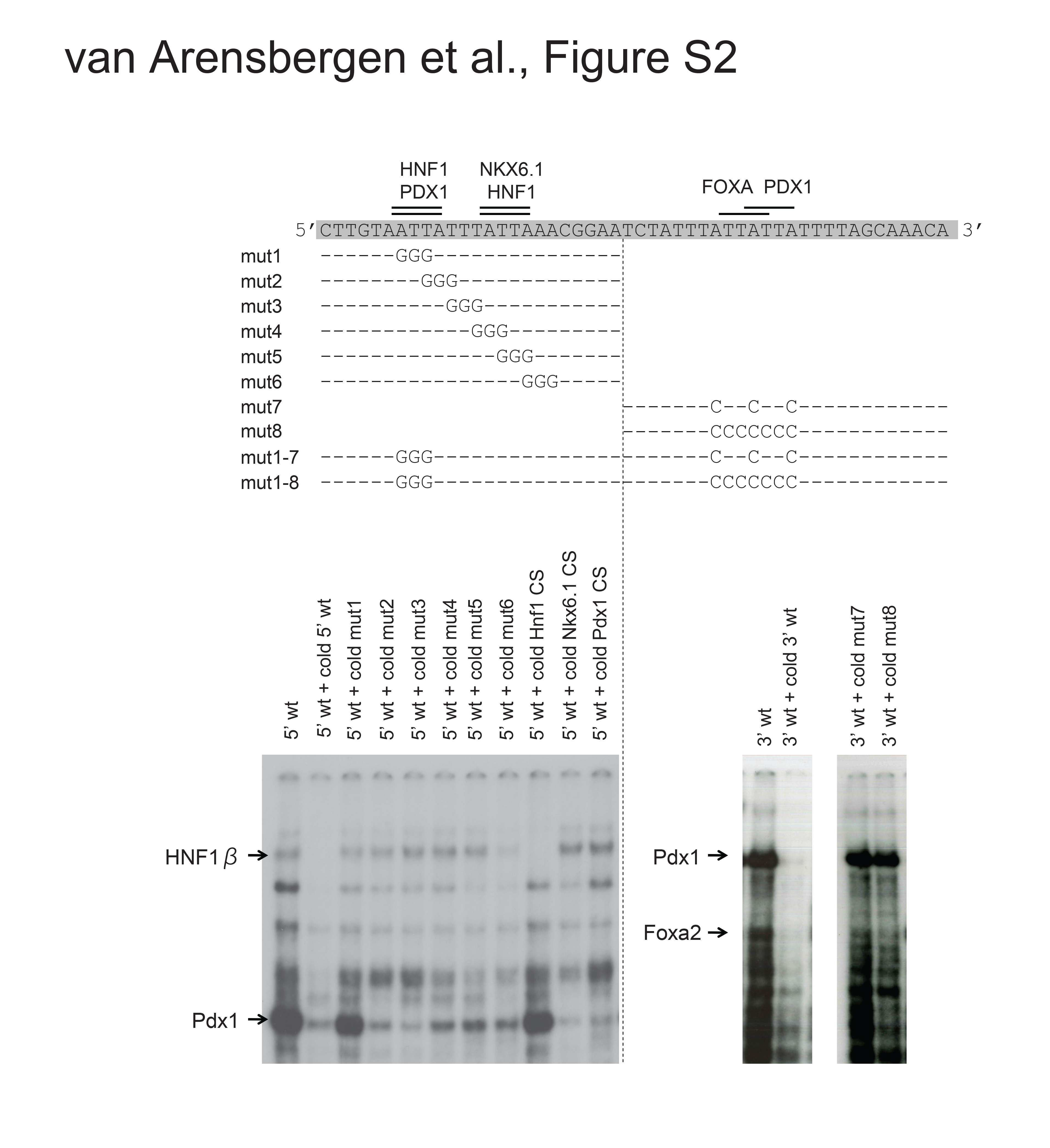

Supplement: S3 Fig — The 5’ part of cis-element 3 (5’WT) was screened with overlapping substitutions of 3 wild-type bases by G nucleotides. The effect of these mutations on DNA binding affinities was assessed by incubating the unlabeled mutant probes at 100-fold excess relative to the wild-type labeled sequence in Min6 nuclear extracts. Note that mutation 1 was found to disrupt both Pdx1 and Hnf1b binding, while Mutation 3 selectively disrupted Hnf1b binding. For the 3’ part of cis-element 3 (3’WT) two mutations were designed and tested in E13.5 pancreatic bud extracts. Both mutations (mut7, mut8) were found to disrupt Pdx1 binding, and mut7 moderately affected Foxa2 binding. (TIF) [file pone.0171508.s003.tif]

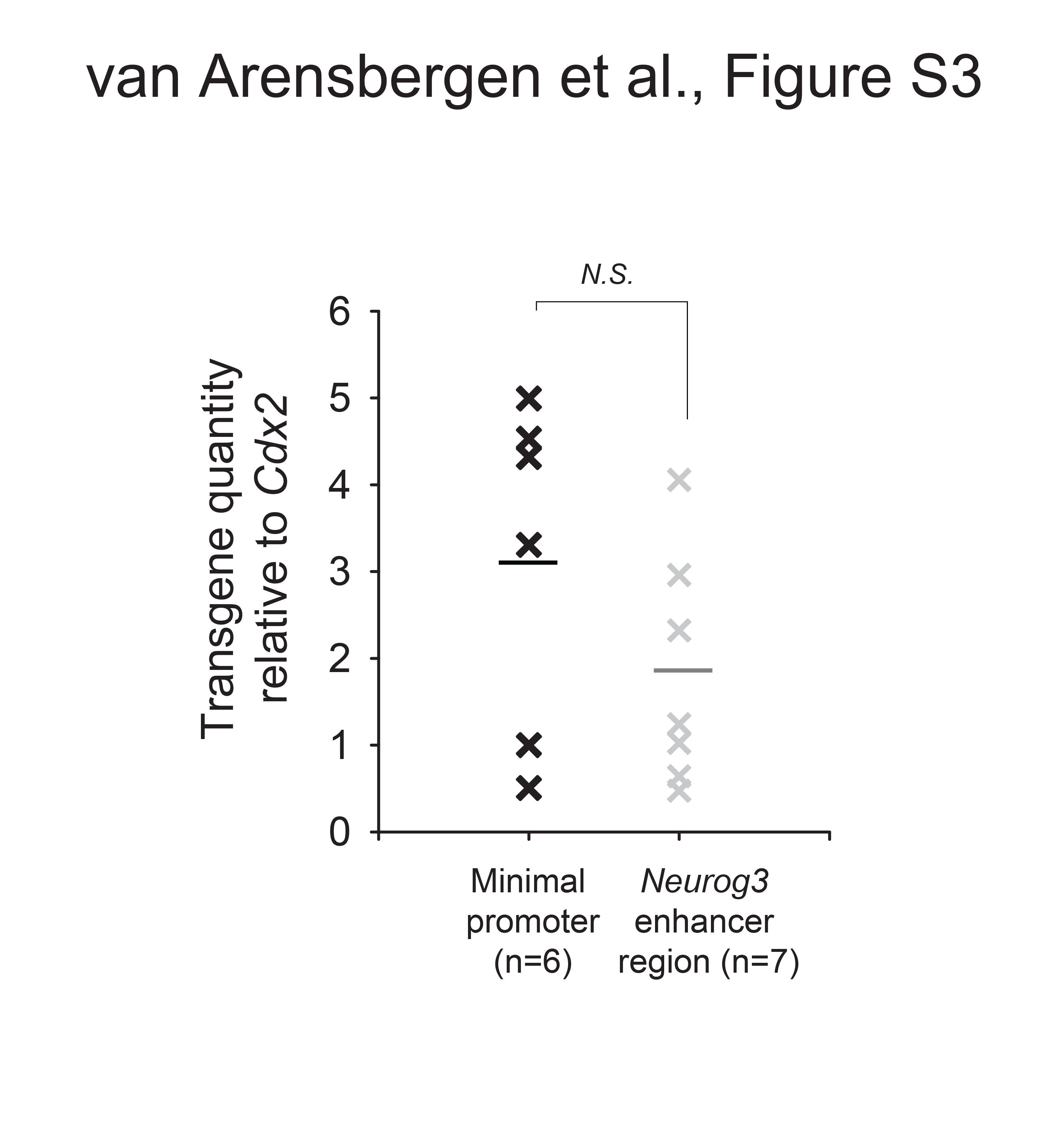

Supplement: S4 Fig — qPCR was performed on genomic DNA isolated from embryonic tails using the GFP 3’UTR primer pair, and Cdx2 was used to normalize the data. Each cross represents an animal. The horizontal lines represent the means. N.S., not significant, Student’s t-test. (TIF) [file pone.0171508.s004.tif]

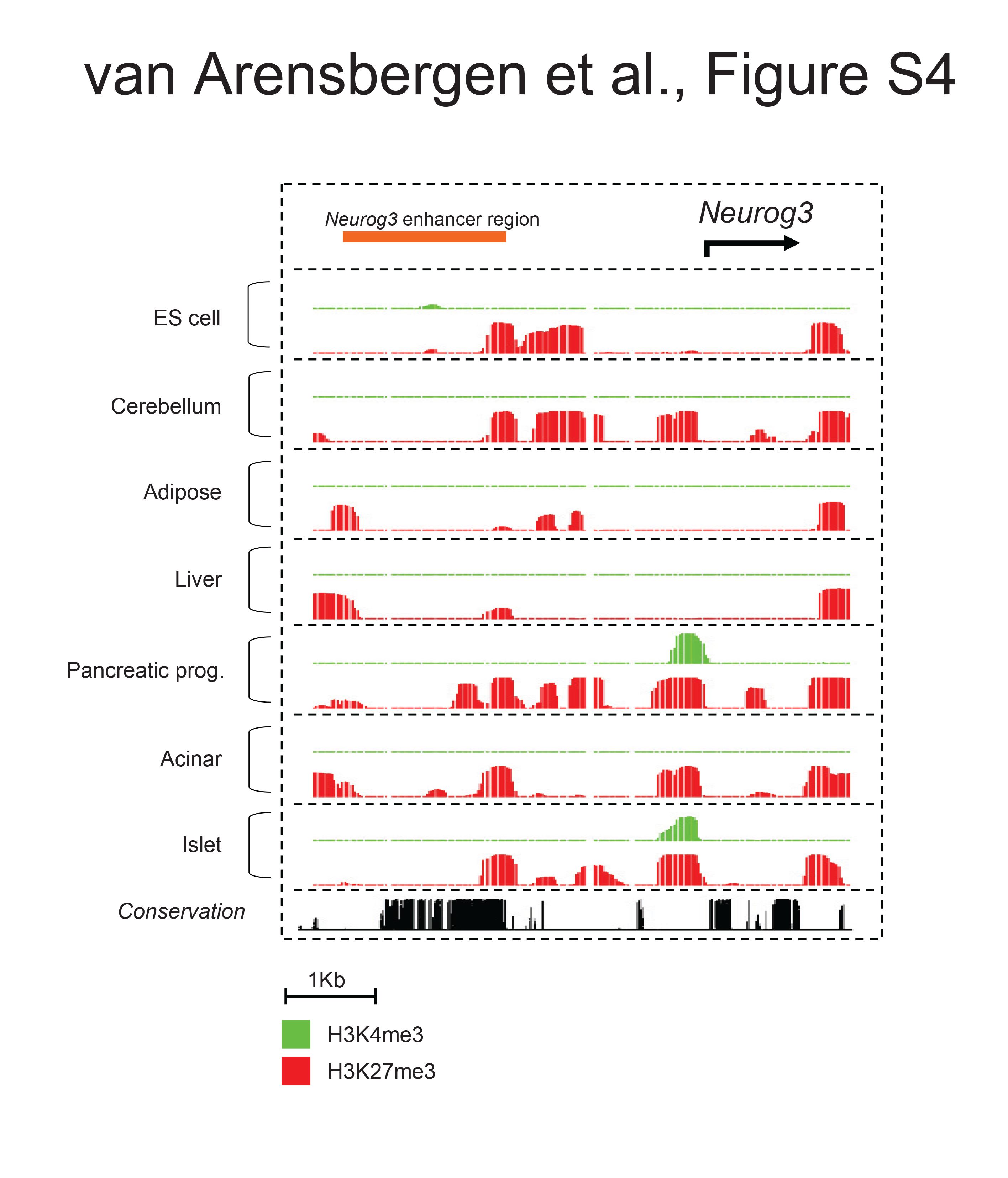

Supplement: S5 Fig — The graph shows the results of an oligonucleotide tiling array analysis of ChIPs for H3K4me3 and H3K27me3 as reported [30]. Enrichment values for H3K4me3 (green) and H3K27me3 (red) are expressed as posterior probability values ranging from 0 to 1. (TIF) [file pone.0171508.s005.tif]

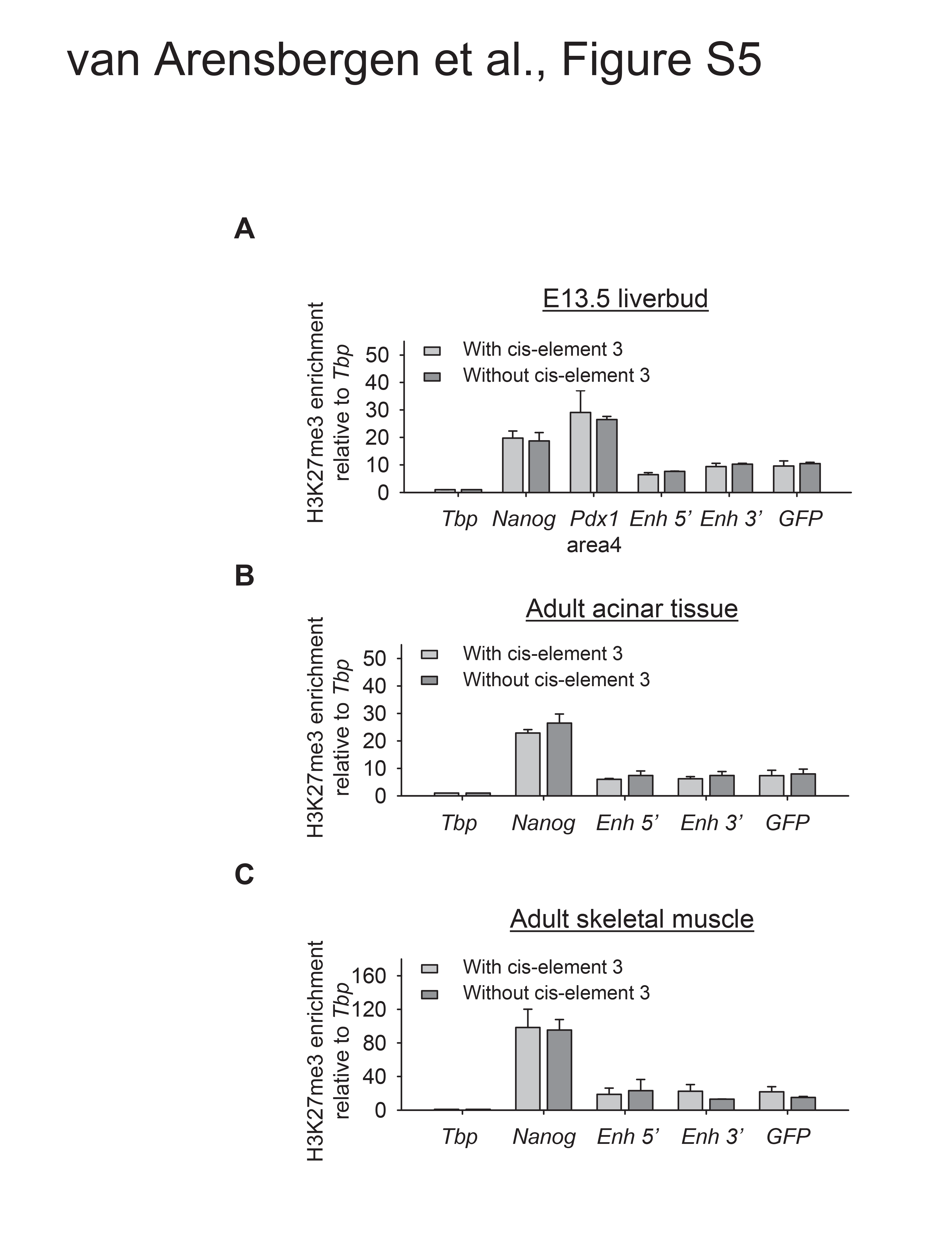

Supplement: S6 Fig — ChIP analysis of H3K27me3 in the exogenous Neurog3 enhancer region with or without cis-element 3 in E13.5 liver (A), adult acinar tissue (B), or adult skeletal muscle (C). (TIF) [file pone.0171508.s006.tif]
