# Supplementary material for: A distal intergenic region controls pancreatic endocrine differentiation by acting as a transcriptional enhancer and as a polycomb response element
Source: PLoS One. 2017 Feb 22;12(2):e0171508. doi: 10.1371/journal.pone.0171508 (PMC5321433; doi:10.1371/journal.pone.0171508)
Supplement: S1 Table — (DOCX) [file pone.0171508.s007.docx]

| **van Arensbergen et al., Supplemental table 1** | |  |
| --- | --- | --- |
|  |  |  |
| **name** | **sequence** | **description** |
|  |  |  |
| WT 5' sense | GATCCTTGTAATTATTTATTAAACGGAA | WT sequence 5' end of Cis-element 3 |
| WT 5' antisense | GATCTTCCGTTTAATAAATAATTACAAG | WT sequence 5' end of Cis-element 3 |
|  | | |
|  |  |  |
| MUT1 sense | GATCCTTGTAGGGATTTATTAAACGGAA | Mutation screen 5' end of Cis-element 3 |
| MUT1 antisense | GATCTTCCGTTTAATAAATCCCTACAAG | Mutation screen 5' end of Cis-element 3 |
| MUT2 sense | GATCCTTGTAATGGGTTATTAAACGGAA | Mutation screen 5' end of Cis-element 3 |
| MUT2 antisense | GATCTTCCGTTTAATAACCCATTACAAG | Mutation screen 5' end of Cis-element 3 |
| MUT3 sense | GATCCTTGTAATTAGGGATTAAACGGAA | Mutation screen 5' end of Cis-element 3 |
| MUT3 antisense | GATCTTCCGTTTAATCCCTAATTACAAG | Mutation screen 5' end of Cis-element 3 |
| MUT4 sense | GATCCTTGTAATTATTGGGTAAACGGAA | Mutation screen 5' end of Cis-element 3 |
| MUT4 antisense | GATCTTCCGTTTACCCAATAATTACAAG | Mutation screen 5' end of Cis-element 3 |
| MUT5 sense | GATCCTTGTAATTATTTAGGGAACGGAA | Mutation screen 5' end of Cis-element 3 |
| MUT5 antisense | GATCTTCCGTTCCCTAAATAATTACAAG | Mutation screen 5' end of Cis-element 3 |
| MUT6 sense | GATCCTTGTAATTATTTATTGGGCGGAA | Mutation screen 5' end of Cis-element 3 |
| MUT6 antisense | GATCTTCCGCCCAATAAATAATTACAAG | Mutation screen 5' end of Cis-element 3 |
|  | | |
|  |  |  |
| WT 3' sense | GATCTCTATTTATTATTATTTTAGCAAACA | WT sequence 3' end of Cis-element 3 |
| WT 3' antisense | GATCTGTTTGCTAAAATAATAATAAATAGA | WT sequence 3' end of Cis-element 3 |
|  | | |
|  |  |  |
| MUT7 sense | GATCTCTATTTCTTCTTCTTTTAGCAAACA | Mutation screen 3' end of Cis-element 3 |
| MUT7 antisense | GATCTGTTTGCTAAAAGAAGAAGAAATAGA | Mutation screen 3' end of Cis-element 3 |
| MUT8 sense | GATCTCTATTCCCCCCCCTTTTAGCAAACA | Mutation screen 3' end of Cis-element 3 |
| MUT8 antisense | GATCTGTTTGCTAAAAGGGGGGGGAATAGA | Mutation screen 3' end of Cis-element 3 |
