# Supplementary material for: A distal intergenic region controls pancreatic endocrine differentiation by acting as a transcriptional enhancer and as a polycomb response element
Source: PLoS One. 2017 Feb 22;12(2):e0171508. doi: 10.1371/journal.pone.0171508 (PMC5321433; doi:10.1371/journal.pone.0171508)
Supplement: S2 Table — (DOC) [file pone.0171508.s008.doc]

**van Arensbergen et al., Supplemental Table 2**

| **Deletion of cis-element 1 and 2** |  |
| --- | --- |
| **External primers** | **Sequence** |
| 5’ external | aatcgtggctggagacagtggca |
| 3’ external | CAGGGAGTCCCTCGAACAAAGGGA |
| **Deletion primers** | **Sequence** |
| Del cis-element 1 sense | AGCCCTTCTCCAAGCTTTCATCTCCTCTGA |
| Del cis-element 1 antisense | TCAGAGGAGATGAAAGCTTGGAGAAGGGCT |
| Del cis-element 2 sense | ATCTCCTCTGACTTCTAGAGGCGAGGAGAGGGA |
| Del cis-element 2 antisense | TCCCTCTCCTCGCCTCTAGAAGTCAGAGGAGAT |
|  |  |
| **Deletion of cis-element 3 and point mutations** |  |
| **External primers** | **Sequence** |
| 5’ -40 M13 | gttttcccagtcacgac |
| 3’ T7 | TAATAGCACTCACTATAGGG |
| **Deletion and point mutation primers** | **Sequence** |
| Del cis-element 3 sense | CCCGCTTTACAATCTAGACTGGGGACAGGTGGG |
| Del cis-element 3 antisense | CCCACCTGTCCCCAGTCTAGATTGTAAAGCGGG |
| Mutation 1 sense | CTTGTA*GGG*ATTTATTAAACGGAA |
| Mutation 1 antisense | ttccgtttaataaaT*ccc*tacaag |
| Mutation 3 sense | CTTGTAATTA*GGG*ATTAAACGGAA |
| Mutation 3 antisense | ttccgtttaat*ccc*taattacaa |
| Mutation 7 sense | cggaatctattt*C*tt*C*tt*C*ttttagcaaaca |
| Mutation 7 antisense | tgtttgctaaaa*G*aa*G*aa*G*aaatagattccg |
