# Supplementary material for: A distal intergenic region controls pancreatic endocrine differentiation by acting as a transcriptional enhancer and as a polycomb response element
Source: PLoS One. 2017 Feb 22;12(2):e0171508. doi: 10.1371/journal.pone.0171508 (PMC5321433; doi:10.1371/journal.pone.0171508)
Supplement: S3 Table — (DOCX) [file pone.0171508.s009.docx]

van Arensbergen et al., Supplemental table 3; Primers ChIP

name description sequence ref.

| Neurog3 -10k FW | primerpair 1 figure 1 | CCTAATGCCATGAGAGGCAGC |  |
| --- | --- | --- | --- |
| Neurog3 -10k RV |  | CTCTCTCTCTGGCCTGTCTAGC |  |
| Neurog3 -6k FW | primerpair 2 figure 1; Neurog3 -6k figure 3 | GCTGCCTTCCAGTCTGGGATAC |  |
| Neurog3 -6k RV |  | GCTGAGATGCAGCAGAGCTTC |  |
| Neurog3 -4199 FW | primerpair 3 figure 1 | ACTTTGTTTCCTGGCTCTGC | Oliver-Krasinski et al., 2009 |
| Neurog3 -4199 RV |  | GTCCTCGAACAAAGGGAGAAG | Oliver-Krasinski et al., 2009 |
| Neurog3 -3332 FW | primerpair 4 figure 1; Neurog3 CE3 figure 3 | AGACTCCCGCCAGAGATGTTT | Oliver-Krasinski et al., 2009 |
| Neurog3 -3332 RV |  | GAAAAGAAAGCCCCACCTGTC | Oliver-Krasinski et al., 2009 |
| Neurog3 -2k FW | primerpair 5 figure 1 | TCTCTGCAGTAGGGAAGGAAT |  |
| Neurog3 -2k RV |  | GAGGAGGGAGCACTACAAAATC |  |
| Neurog3 TS FW | primerpair 6 figure 1 | GGCACCCCCAAACCTCCTTC |  |
| Neurog3 TS RV |  | TCTGTTTGTTCCCCCACTCCAC |  |
| Pdx1 area 4 | primerpair to a known enhancer | TTGTTGTGTCGCTAGCTGGTCTGT |  |
| Pdx1 area 4 |  | TGCTCTGGGCTCTGGCTTATTTAC |  |
| Des6 FW | primerpair in gene desert on chr.6 | TCTCATGACCTTGACATCTAGGGC |  |
| Des6 RV |  | TGAAGACACGGATGTGCTTCTAAC |  |
| GFP FW | GFP coding region | CCTGAAGTTCATCTGCACCACC |  |
| GFP RV |  | TCGTGCTGCTTCATGTGGTC |  |
| Enh. 5' FW | specific for 5' region of exogenous neurog3 enhancer | GGACAGCAGAGATCCACTTTGG |  |
| Enh. 5' RV |  | AGGAAGACGCTATGGGGGAC |  |
| Enh. 3' FW | specific for 3' region of exogenous neurog3 enhancer | CAAAGAACCCCCAGGCAATGC |  |
| Enh. 3' RV |  | AGCAATAGATGGCTCTGCCCTG |  |
| Tbp TSS | used for ChIP normalization | ATCAGATGTGCGTCAGGCGTT |  |
| Tbp TSS |  | TGCGGAGAAAATGACGCGA |  |
| Nanog TSS | used as negative control in ChIP | AGAGGATGCCCCCTAAGCTTT |  |
| Nanog TSS |  | ACAGTTAATCCCACCTGCAGG |  |

van Arensbergen et al., Supplemental table 3; Primers RT-PCR and genotyping

name sequence description

| eGFP FW GACCACATGAAGCAGCACGACTTCT eGFP RV TTCTGCTGGTAGTGGTCGGCGAGCT | | primers to genotype transgenics |
| --- | --- | --- |
| GFP 3'UTR (LTR) FW GGCAGCTGTAGATCTTAGCCACTT  GFP 3'UTR (LTR) RV CTTCGTTGGGAGTGAATTAGCCCT | | qPCR primers for quantitative genotyping and qRT-PCR |
| Cdx2 FW | GCCAGGGACTATTCAAACTACAGG | qPCR primers for normalization of quantitative genotyping |
| Cdx2 RV | GACTTCGGTCAGTCCAGCTATCTT |  |
| Tbp FW | ACCCTTCACCAATGACTCCTATG | qPCR primers for normalization in qRT-PCR |
| Tbp RV | ATGATGACTGCAGCAAATCGC |  |
